# No psychological distress in sportsmen aged 45 years and older after cardiovascular screening, including cardiac CT: The Measuring Athlete’s Risk of Cardiovascular events (MARC) study

**DOI:** 10.1007/s12471-017-0948-5

**Published:** 2017-01-31

**Authors:** M. M. Schurink, T. L. Braber, N. H. J. Prakken, P. A. F. M. Doevendans, F. J. G. Backx, D. E. Grobbee, R. Rienks, H. M. Nathoe, M. L. Bots, B. K. Velthuis, A. Mosterd

**Affiliations:** 1 Department of Radiology, University Medical Center Utrecht, Utrecht, The Netherlands; 2 Department of Cardiology, University Medical Center Utrecht, Utrecht, The Netherlands; 3 Department of Cardiology, Meander Medical Center Amersfoort, Amersfoort, The Netherlands; 4 Department of Radiology, University Medical Center Groningen, Groningen, The Netherlands; 5 Department of Rehabilitation, Nursing Science and Sports, University Medical Center Utrecht, Utrecht, The Netherlands; 6 Julius Center for Health Sciences and Primary Care, University Medical Center Utrecht, Utrecht, The Netherlands

**Keywords:** Athletes, Screening, Coronary computed tomography angiography (CCTA), Psychological stress, Sports

## Abstract

**Background:**

Psychological distress caused by cardiovascular pre-participation screening (PPS) may be a reason not to implement a PPS program. We assessed the psychological impact of PPS, including cardiac computed tomography (CT), in 318 asymptomatic sportsmen aged ≥45 years.

**Methods:**

Coronary artery disease (CAD) was defined as a coronary artery calcium score ≥100 Agatson units and/or ≥50% luminal stenosis on contrast-enhanced cardiac CT. Psychological impact was measured with the Impact of Event Scale (IES) (seven items) on a six-point scale (grade 0–5). A sum score ≥19 indicates clinically relevant psychological distress. A Likert scale was used to assess overall experiences and impact on sports and lifestyle.

**Results:**

A total of 275 participants (86.5% response rate, 95% CI 83–90%) with a mean age of 54.5 ± 6.4 years completed the questionnaires, 48 (17.5%, 95% CI 13–22%) of whom had CAD. The median IES score was 1 (IQR 0–2, [0–23]). IES was slightly higher in those with CAD (mean rank 175 vs. 130, *p* < 0.001). One participant (with CAD) experienced clinically relevant psychological distress (IES = 23). Participants reported numerous benefits, including feeling safer exercising (58.6%, 95% CI 53–65%) and positive lifestyle changes, especially in those with CAD (17.2 vs. 52.1%, *p* < 0.001). The majority were satisfied with their participation (93.8%, 95% CI 91–97%).

**Conclusion:**

Cardiovascular PPS, including cardiac CT, causes no relevant psychological distress in older sportsmen. Psychological distress should not be a reason to forego screening in sportsmen.

**Electronic supplementary material:**

The online version of this article (doi: 10.1007/s12471-017-0948-5) contains supplementary material, which is available to authorized users.

## Introduction

Regular physical exercise reduces the risk of cardiovascular disease, [1, 2] but vigorous exertion (particularly in untrained persons) can trigger an acute cardiac event [3, 4]. This phenomenon is known as the paradox of sports. Exercise-related cardiac arrest is the leading cause of mortality during exercise [5]. Over 90% of these arrests occur in men aged ≥45 years, with coronary artery disease (CAD) as the major cause [6, 7]. In the Netherlands, the incidence of exercise-related cardiac arrest in men >35 years is 5.8 per 100,000 per year, with approximately 50% surviving the event [7]. The increasing popularity of sports, especially in middle-aged and older individuals, is likely to lead to an increase in exercise-related arrests [8, 9].

Some countries (though not the Netherlands) have adopted the policy of mandatory pre-participation screening (PPS) of young (≤35 years) competitive athletes [10]*. *Increasingly, older sportsmen voluntarily undergo a preventive sports medical examination [9]. Recommendations regarding sports medical examinations of senior sportsmen have been published by the European Society of Cardiology (ESC), with the main objective of ruling out significant occult CAD [9, 11].

As the merits of PPS are still under debate, [12] recommendations vary across countries, age groups, sports disciplines and competition levels [13]. An ideal screening program meets the following six criteria: 1) the condition must have a significant impact on public health and 2) should have an asymptomatic period during which detection may be possible, 3) the outcome for a condition should improve by treatment during this asymptomatic period, 4) the screening test should be sensitive enough to detect the disease during the asymptomatic period, 5) specific enough to minimise false-positive results and 6) acceptable to those undergoing the test [14]. The US National Heart, Lung, and Blood Institute outlined the need to understand the psychological burden of screening in athletes, prior to widespread implementation of the PPS program [15]. Many physicians hesitate to embark on large-scale PPS, citing psychological distress caused by the screening and the potential outcome as an important consideration.

To date, only two studies on the psychological impact of PPS have been performed [16, 17]. Both were carried out in relatively young persons (mean age 16 (*n* = 952) and 26 years (*n* = 441)), whose PPS included medical history and physical examination, combined with a resting electrocardiogram (ECG) (*n* = 917) or echocardiography (*n* = 441) [16, 17]. Screening caused no relevant psychological distress in these groups. The psychological impact of PPS in those most frequently affected by exercise-related cardiac arrests, sportsmen aged 45 years and older, remains to be investigated. The aim of this study was to determine the psychological impact of cardiovascular screening, including cardiac computed tomography (CT), in asymptomatic recreational sportsmen aged 45 years or over.

## Methods

The design and main results of the Measuring Athlete’s Risk of Cardiovascular events (MARC) study have been published [18, 19]. The study has been approved by the regional medical ethics committee. In brief, asymptomatic middle-aged (≥45 years) sportsmen whose routine sports medical examination (including medical history, physical examination, resting and bicycle exercise ECG) revealed no cardiac abnormalities were eligible to undergo additional cardiac CT imaging (mean radiation dose 3.9 mSv) The presence of relevant CAD was defined as a coronary artery calcium score (CACS) ≥100 Agatston units (AU) on non-contrast coronary CT and/or ≥50% luminal stenosis on contrast-enhanced coronary CT angiography. Cardiac CT identified occult CAD in 60 (18.9%, 95% CI 14.9–23.5%) of the 318 participants, resulting in a five-year estimated number-needed-to-screen of 159 (95% CI 128–201) to prevent one cardiovascular event with statin treatment [19]. All 318 MARC participants were invited by email to fill out an internet-based questionnaire to evaluate the psychological impact of participating, with an interval of 7–30 (mean 16) months after undergoing cardiac CT. A total of three reminders were sent to those who did not respond.

Similar to Solberg et al. [16] , we used the intrusion sub-scale of the Impact of Event Scale (IES). This scale was originally developed to measure posttraumatic stress [20, 21]. The seven items of the IES were graded on a six-point scale (0 = never, 1 = a little, 2 = somewhat, 3 = medium, 4 = much, 5 = very much). A sum score ≥19 is generally accepted to indicate clinically relevant psychological distress [22]. In addition, the questionnaire contained items concerning global experiences and impact on sports and lifestyle, measured on a Likert scale (1 = strongly disagree, 2 = disagree, 3 = neutral, 4 = agree and 5 = strongly agree). The questionnaires were identical for all, except for one additional question (regarding advice received on sporting activities) that was added for those found to have relevant CAD (see online Supplementary Data).

### Statistical analysis

The primary outcome (IES score) was calculated as median with its interquartile range (IQR). Secondary outcomes (general experiences, impact on sports and lifestyle) were dichotomised and reported in frequencies and percentages. Comparisons between those with and without CAD were conducted with the Mann-Whitney U test or Chi-square test. Data analysis was performed using SPSS statistics (version 22.0 SPSS Inc. Chicago, Illinois). A *p*-value <0.05 was considered statistically significant.

## Results

**Fig. 1:**
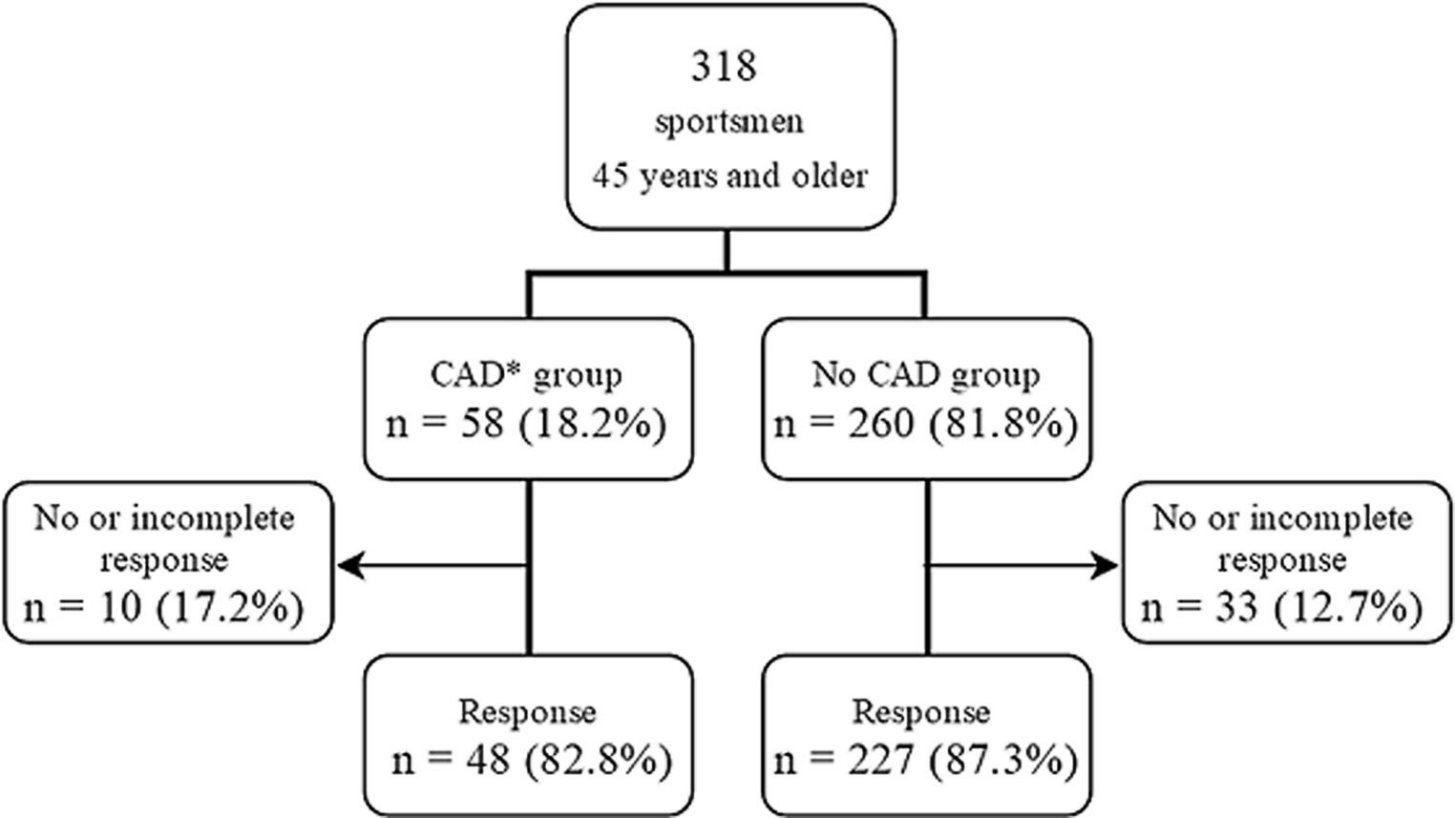
Flow diagram of participants with an overall response rate of 86.5% (95% CI 83–90%). (*CAD* coronary artery disease. CAD* group coronary artery calcium scoring (CACS) ≥100 AU on non-contrast CCT and/or ≥50% luminal stenosis on contrast-enhanced CCTA)

The questionnaire was completed by 275 of the 318 MARC participants with a mean age of 54.5 ± 6.4 years (Fig. 1). Altogether 48 of 58 participants with CAD and 227 of 260 participants without CAD responded (82.8%, 95% CI 73–93 and 87.3%, 95% CI 83–91% response rate, respectively). The response rate did not differ between these groups (*p* = 0.361). The characteristics of the study population are shown in Table 1. Participants were asymptomatic and almost all (96%) had a low cardiovascular risk (ESC Systematic COronary Risk Evaluation (SCORE) 0–4%). All participants were Caucasian and they were fit, as evidenced by a mean maximal exercise capacity of 314 W. They were predominantly engaged in cycling (45%) and long distance running (36%). The main reason to participate in the MARC study was cardiac screening in the context of healthy and safe sports (43%). Only 4% had concerns about their own cardiac condition.

**Table 1:** Baseline characteristics

	All *n* = 275	CAD *n* = 48 (17%)	No CAD *n* = 227 (83%)	*p*-value
Age (years)	54.5 ± 6.4	57.9 ± 6.1	53.8 ± 6.3	<0.001*
Height (m)	1.82 ± 0.07	1.81 ± 0.07	1.83 ± 0.07	0.119
Weight (kg)	82.6 ± 10.4	84.5 ± 11.1	82.2 ± 10.2	0.163
BMI (kg/m²)	24.8 ± 2.6	25.9 ± 3.1	24.6 ± 2.5	0.003*
Systolic blood pressure (mm Hg)	129 ± 13	131 ± 13	129 ± 14	0.216
Diastolic blood pressure (mm Hg)	80 ± 9	82 ± 7	80 ± 9	0.122
History of hypertension, *n* (%)	15 (5.5)	6 (12.5)	9 (4.0)	0.018*
Current smoker, *n* (%)	8 (2.9)	1 (2.1)	7 (3.1)	0.709
Former smoker, *n* (%)	93 (33.8)	24 (50)	69 (30.4)	0.009
Diabetes mellitus, *n* (%)	2 (0.7)	0 (0)	2 (0.9)	0.516
Family history of CAD, *n* (%)	123 (44.7)	26 (54.2)	97 (42.7)	0.149
Total cholesterol (mmol/l)	5.4 ± 0.8	5.7 ± 0.9	5.3 ± 0.8	0.002*
*ESC SCORE risk categories*				
Low (0–4%), *n* (%)	264 (96)	44 (92)	220 (97)	0.092
Intermediate (5–9%), *n* (%)	11(4)	4 (8)	7 (3)	0.092
High (≥10%), *n* (%)	0	0	0	–
*Exercise tolerance*				
Total Watt	314 ± 48	304 ± 49	316 ± 47	0.117
Watt/kg	3.8 ± 0.7	3.6 ± 0.6	3.9 ± 0.7	0.015*
*Motivation to participate, n (%)*				
General screening	118 (42.9)	23 (47.9)	95 (41.8)	0.442
Contribution to science	77 (28.0)	8 (16.7)	69 (30.4)	0.055
Relatives with cardiac disease	36 (13.1)	9 (18.8)	27 (11.9)	0.202
Concerns regarding cardiac condition	12 (4.4)	3 (6.2)	9 (4.0)	0.483
Other	32 (11.6)	5 (10.4)	27 (11.9)	0.773

*Caption*: Data are presented as mean ± SD, proportions (%) or median values [IQR]

*BMI* body mass index; *CAD* coronary artery disease; *ECG* electrocardiogram

Risk factors were defined by chart review, including review of medications for hypertension, lipid disorders or diabetes

*significant difference (*p* < 0.05) between CAD and no CAD group.

All participants diagnosed with CAD (*n* = 58 of 318, 18.2%, 95% CI 14–23%) received lifestyle advice, were encouraged to continue their sports activities, but advised to avoid excessive/peak efforts, and suggested to contact their general practitioner to consider initiating statin treatment. The minority (*n* = 17) with severe CAD (CACS ≥400 AU and/or ≥50% coronary artery stenosis) were advised to consult a cardiologist. Thirteen participants underwent additional cardiac testing (myocardial adenosine perfusion imaging (*n* = 9) or coronary angiography (*n* = 4)), resulting in a percutaneous coronary intervention in four of them.

**Fig. 2:**
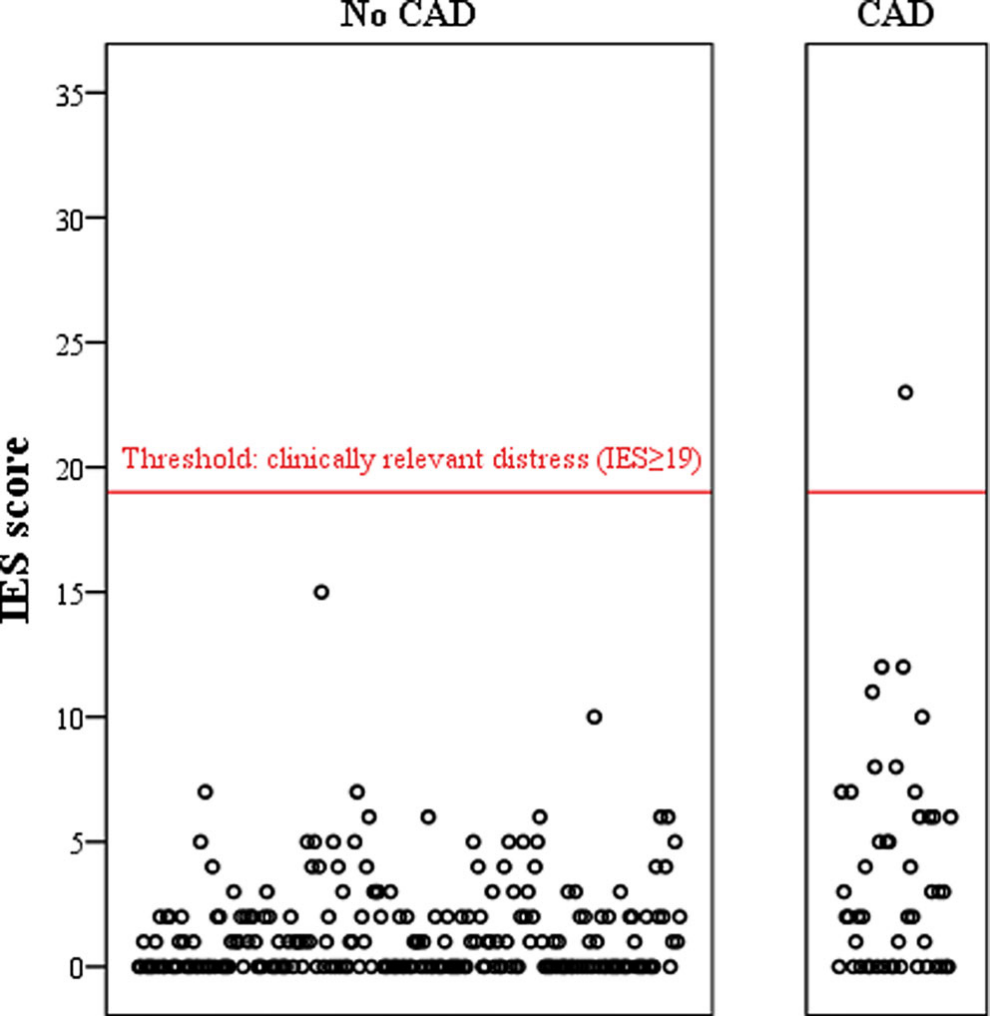
Impact of event scores according to presence of coronary artery disease. (*IES score* impact of event score, *CAD* coronary artery disease. Each dot represents an individual MARC study participant)

The median IES score of participants was 1 (IQR 0–2), obtained on average 16 months (range 7 to 30 months) post screening. The IES in participants with CAD was significantly higher than in those without CAD (median 1 vs. 2, mean rank 175 vs. 130, *p* < 0.001). Only one participant (with CAD) experienced clinically relevant psychological distress, defined as IES ≥19 (Fig. 2).

**Fig. 3:**
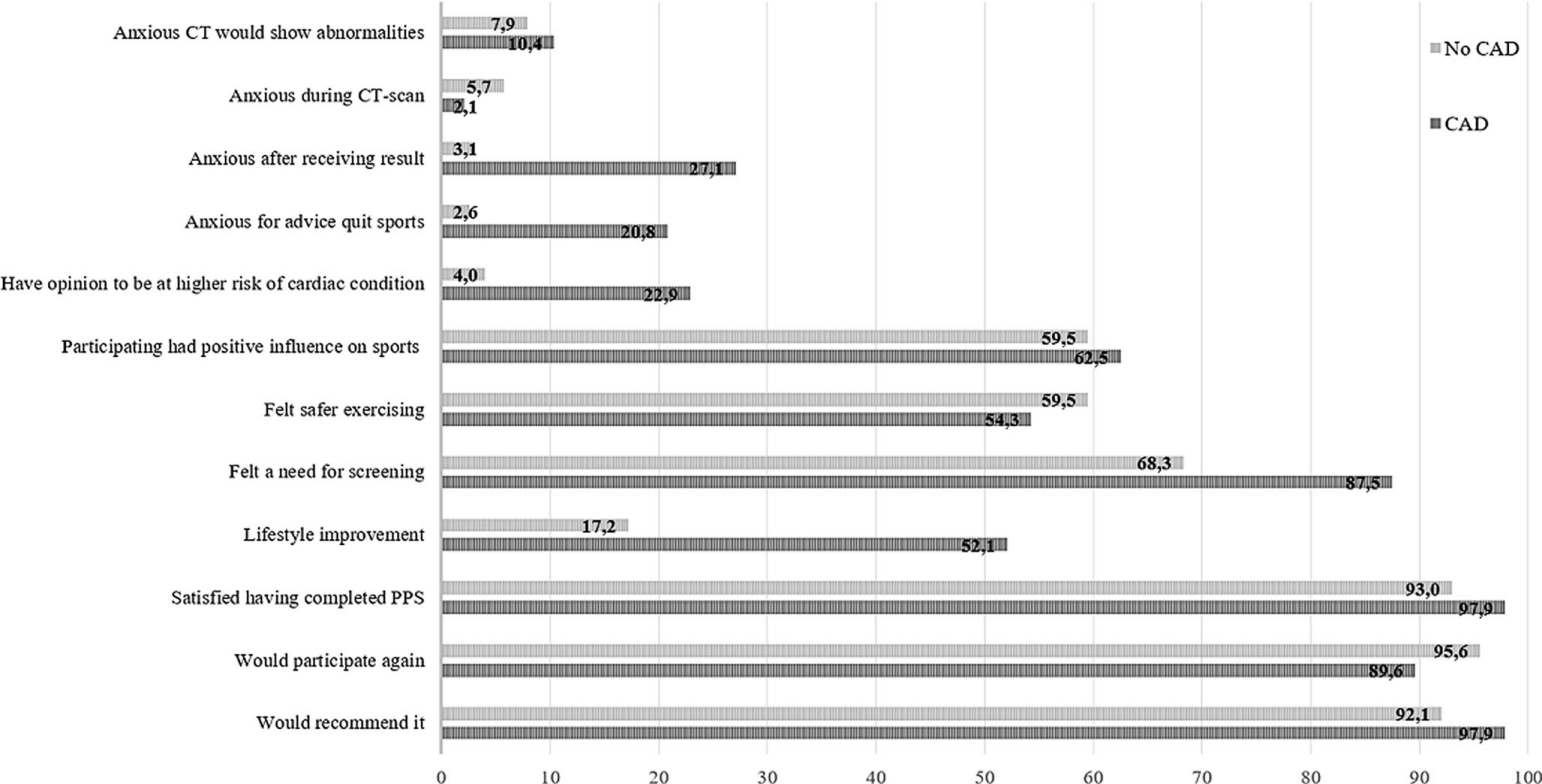
Perspectives about pre-participation screening with cardiac CT (in %)

The personal and general perspectives about PPS including cardiac CT are shown in Fig. 3. Relatively few participants experienced anxiety before (8%, 95% CI 5–12%) or during (5%, 95% CI 2–8%) CT scanning, and no significant differences were seen between participants with or without CAD. Participants found to have CAD were more likely to feel anxious directly after receiving the result (27.1% vs. 3.1%, *p* < 0.001), to be afraid they would be advised to quit sports (20.8% vs. 2.6%, *p* < 0.001) and to have the opinion they were at higher risk of a cardiac condition than other sportsmen (22.9% vs. 4.0%, *p* < 0.001).

In general the screening had a positive influence on sporting activities, only 15 participants (5.5%, 95% CI 3–8%) disagreed and 34.5% (95% CI 29–40%) had a neutral opinion. The majority (58.6%, 95% CI 53–65%) felt safer exercising, whereas 32% (95% CI 26–38%) experienced no difference and a minority (9.4%, 95% CI 6–13%) felt less safe exercising. No significant differences were observed between the two groups for these two items.

Those found to have CAD more frequently felt a need for PPS screening, including CT imaging, of middle-aged sportsmen than those without CAD (87.5% vs. 68.3%, *p* = 0.007) (Fig. 3). Participating in the MARC study led to lifestyle improvement in 64 sportsmen (23.3%), predominantly in those found to have CAD (17.2 vs. 52.1%, *p* < 0.001). The majority of these 64 participants (65.6%, 95% CI 54–77%) adjusted to a healthier diet, 46.8% (95% CI 35–59%) lost weight, 35.9% (95% CI 24–48%) increased relaxing and/or quality time and 6.2% (95% CI 0–12%) quit smoking.

In the end, the vast majority were satisfied with their participation (93.8%, 95% CI 91–97%), would participate again (94.5%, 95% CI 92–97%) and would recommend participation to others (93.1%, 95% CI 90–96%). These opinions did not differ between those with and without CAD (*p* = 0.194, 0.096 and 0.147, respectively).

## Discussion

We found no relevant psychological distress in asymptomatic middle-aged recreational sportsmen who underwent cardiovascular PPS with cardiac CT, irrespective of whether they were found to have CAD or not. The median IES score, assessed on average 16 months after the cardiac CT, was slightly higher in the participants with CAD, but did not reach the threshold of clinical relevance. Transient anxiety, directly after being informed about the results, was significantly more frequent in those diagnosed with CAD. Nearly all were satisfied with their participation and would recommend PPS to others. Participants reported numerous benefits, including feeling safer exercising and positive lifestyle changes, especially in those with CAD.

Our study confirms the results of two earlier studies that found no association between cardiovascular screening and psychological distress in younger athletes, [16, 17] and extends this observation to middle-aged recreational sportsmen who underwent cardiac CT (with a mean radiation dose of 3.9 mSv) in addition to the routine sports medical examination.

The psychological consequences of screening asymptomatic persons have been evaluated in other domains. A meta-analysis of 12 studies (*n* = 170,045, mean age varying from 41 to 69 years) documented no significant impact of screening for cancer, diabetes, abdominal aortic aneurysm, osteoporosis, peptic ulcer or coronary artery disease on anxiety, depression or quality of life, not even in those receiving positive test results [23]. A prospective investigation among 685 men aged 65 to 73 years screened for abdominal aortic aneurysm found transient psychological stress with a small decrease in overall quality of life when offered the possibility to be screened [24]. Overall, those screened reported a better quality of life compared with controls (non-screened), although being diagnosed with an aneurysm did impair quality of life. This suggests that the results of screening rather than the procedure cause stress [24]. It follows that screening programs should have support mechanisms for individuals with a positive result.

This is the first study investigating the psychological impact of an extensive cardiovascular PPS test that included cardiac CT in asymptomatic men aged ≥45 years. Stress was measured with a frequently used and validated tool (IES questionnaire) [25]. The response rate was high (86.5%, 95% CI 83–90) and the characteristics of participants were essentially similar to those of the complete MARC cohort. The percentage (17.5%) of participants with a positive result (CAD) was high compared with two former studies of psychological distress caused by PPS (5% and 0.7%, respectively) [16, 17].

This study has limitations. First, the psychological assessment was performed 7–30 months post screening. Although this provides insight into the long-term effects of screening, it may also have led to recall bias affecting the short-term psychological impact, because normally only extreme experiences will be remembered well. Second, as our participants were all Caucasian men who participated on a voluntary basis, the results cannot readily be extrapolated to the larger group of older athletes. The IES score is probably lower than it would have been if participants had undergone mandatory screening. Also, the response to stress and coping mechanisms are likely to differ between men and women [26].

Third, the reassuring knowledge that the routine sports medical examination revealed no cardiac abnormalities may have blunted the psychological impact of cardiac CT. Fourth, as we did not randomise to PPS or no PPS, we have no information from a control group.

We do not advocate mandatory PPS at this stage [27]. Although the addition of cardiac CT to the sports medical examination does not have a major psychological impact in older sportsmen, a randomised study is needed to investigate whether the introduction of a sports medical examination (including CT scanning) reduces the incidence of (exercise-related) cardiac events in older athletes and is cost-effective.

In conclusion, PPS with cardiac CT, both coronary artery calcium scoring and coronary CT angiography, causes no relevant long-term psychological distress in recreational Caucasian sportsmen aged 45 years and older. Participants reported numerous benefits, including feeling safer exercising (58.6%, 95% CI 53–65%) and positive lifestyle changes, especially in those with CAD (17.2 vs. 52.1%, *p* < 0.001). The majority of participants were satisfied and would recommend the evaluation to others. Psychological distress should not be a reason to forego screening in older sportsmen. However, attention to transient anxiety in those with a positive result (CAD) is needed.

## Caption Electronic Supplementary Material

Questionnaire
