# Supplementary material for: No psychological distress in sportsmen aged 45 years and older after cardiovascular screening, including cardiac CT: The Measuring Athlete’s Risk of Cardiovascular events (MARC) study
Source: Neth Heart J. 2017 Jan 31;25(4):271–7. doi: 10.1007/s12471-017-0948-5 (PMC5355386; doi:10.1007/s12471-017-0948-5)
Supplement: Supplementary file 1 — Questionnaire [file 12471_2017_948_MOESM1_ESM.docx]

**SUPPLEMENTARY MATERIAL**

**Questionnaire**

**A. Impact of Event Scale**

Below you find some sentences that are used by persons who have experienced strain. These sentences describe how they feel after undergoing an event that has had an impact on them. Read each sentence and tick the number 0 to 5 corresponding to how you remember feeling in the period after the heart screening in the context of MARC study.

|  | Never  0 | A little  1 | Some what  2 | Medium  3 | Much  4 | Very much  5 |
| --- | --- | --- | --- | --- | --- | --- |
| Q1.  I have had periods with strong emotions regarding the heart investigation. | □ | □ | □ | □ | □ | □ |
| Q2.  Things I have heard or seen suddenly remind me of the heart screening. | □ | □ | □ | □ | □ | □ |
| Q3.  I have started thinking of the heart screening when I have not wanted so. | □ | □ | □ | □ | □ | □ |
| Q4.  Images of the heart screening have suddenly appeared in my mind. | □ | □ | □ | □ | □ | □ |
| Q5.  Any reminder regarding the heart screening has evoked feelings in me. | □ | □ | □ | □ | □ | □ |
| Q6.  I have had sleeping problems due to thoughts and images from the heart screening. | □ | □ | □ | □ | □ | □ |
| Q7.  I have had bad dreams about the heart screening. | □ | □ | □ | □ | □ | □ |

**B. Experiences**

Directions: for the following questions, please choose the box that best describes your opinion.

|  | Strongly disagree  1 | Disagree  2 | Neither agree nor disagree  3 | Agree  4 | Strongly agree  5 | Not applicable  - |
| --- | --- | --- | --- | --- | --- | --- |
| Q1.  I was satisfied with my participation in the study in general | □ | □ | □ | □ | □ |  |
| Q2.  While undergoing the CT-scan, I felt anxious | □ | □ | □ | □ | □ |  |
| Q3.  I was afraid that the CT-scan would reveal abnormalities of my heart. | □ | □ | □ | □ | □ |  |
| Q4.  Immediately after I received my final results, I felt anxious. | □ | □ | □ | □ | □ |  |
| Q5.  I was afraid someone would give me the advice to stop play sports. | □ | □ | □ | □ | □ |  |
| Q6.  Compared to other heart screens that I have had, I feel safer playing sports  . | □ | □ | □ | □ | □ | □ |
| Q7.  I would participate again in similar research. | □ | □ | □ | □ | □ |  |
| Q8.  I feel that all athletes of ≥45 years should receive a sport medical evaluation including CT-scan. | □ | □ | □ | □ | □ |  |
| Q9.  I would recommend this heart screen to other athletes. | □ | □ | □ | □ | □ |  |
| Q10.  Compared to other athletes, I think that I am more likely to have a heart condition in the future. | □ | □ | □ | □ | □ |  |
| Q11.  The impact of this heart screen on my training/competition was positive. | □ | □ | □ | □ | □ |  |

**C. Motivation**

Q19. What was your main motivation to participate in this study?

□ Worries about health state of my heart

□ Heart disease or -symptoms in family or friends

□ Check to healthy and safe sports

□ Contribute to science

□ Otherwise

**D. Follow up**

Q20. Did your results lead to an advice to restrict sporting activities ? * (only for those found to have CAD)

□ No

□ Yes, namely …

Q21. Do you play sports more often since your participation in the MARC study?

□ Yes

□ No, as much as before

□ No, less than before

Q22. Did you change your lifestyle into a healthier pattern since your participation in the MARC study?

□ Yes, I adjusted a healthier diet

□ Yes, I lost weight

□ Yes, I quit smoking

□ Yes, I try to relax better and/or more

□ No, I did not change my lifestyle

Q23. Did you have a cardiovascular event (e.g. myocardial infarction, cerebrovascular accident or TIA) or other reason for admission to hospital, since your participation in the MARC study?

□ No

□ Yes, namely …

Q24. Did you get a blood pressure control since your participation?

□ No

□ Yes, namely … / … mmHg

Q25. Did you get a cholesterol control since your participation?

□ No

□ Yes, namely …

Q26. Did you quit smoking since your participation?

□ No, I am still smoking

□ Yes

□ Inapplicable (I am a non-smoker)

Q27. What is your current weight?

…kg

Q28. Do you use medication?

□ No

□ Yes, namely … (medication, dose)

29. Any comments and suggestions?
